# Reply to: Is muscular strength compromised by overnight fasting or food ingestion in hospital settings?

**DOI:** 10.31744/einstein_journal/2019CE5406

**Published:** 2019-11-13

**Authors:** Wesley Santana Correa-Arruda, Iara dos Anjos Vaez, José Eduardo de Aguilar-Nascimento, Diana Borges Dock-Nascimento

**Affiliations:** 1 Universidade Federal de Mato Grosso Graduate Program in Health Sciences, Universidade Federal de Mato Grosso, Cuiabá, MT, Brazil.

Initially, we are grateful for the considerations made by Rodrigues et al. The central idea of the study was to investigate if overnight fasting, often required for surgical or diagnostic procedures, has an impact on muscle function. Doubtless, the findings showed that the 8-hour night fasting, although physiological, resulted in decreased functional capacity determined by dynamometry. This is aggravated by the fact that fasting for procedures in Brazilian hospitals, prescribed for 8 hours, is really longer, and is estimated at up to 18 hours, on average, sometimes reaching 24 hours, in some cases.^1^ This is relevant, and the findings showed that muscle function may be hindered when this prolonged fasting is during the day,^2^ considering that the patient is awake and in greater metabolic stress.^3^ Another important point in our findings is that muscle strength increased according to the quantity of the evening meal consumed the night before the measurement of strength and after breakfast and lunch. This showed a positive interference of the ingestion of calories and nutrients on muscle function. These results reinforced the need to guarantee that the patients ingest or receive, via nutritional therapy, all their nutritional needs,^4^ and that the fasting time for procedures, tests, or surgeries, as per recommendation of the ACERTO project,^5^be reduced.^6^ Thus, just as fasting may result in reduced muscle strength, leading to worse clinical outcomes,^7^ and the ingestion of food improved this muscle capacity, in daily routine, it is necessary to reduce fasting time and guarantee a greater ingestion of calories and nutrients to the patients. The inclusion of tables and figures aimed only to give the readers a quicker view of the findings which, as you yourselves recorded, are relevant. Finally the odds ratio also gives the reader an idea of the chance of an event occurring, and this adds information − oftentimes, greater than only the p value. Hence, we believe the conclusions were consistent with the objectives established and the results obtained. We hope these considerations help the authors and the readers better understand the original article published by the journal einstein.
