# Supplementary material for: Reply to: Is muscular strength compromised by overnight fasting or food ingestion in hospital settings?
Source: Einstein (Sao Paulo). 2019 Nov 13;17(4):eCE5406. doi: 10.31744/einstein_journal/2019CE5406 (PMC6855698; doi:10.31744/einstein_journal/2019CE5406)
Supplement: Translation

# Translation: Resposta para: A força muscular é comprometida pelo jejum noturno ou pelo percentual de ingestão da dieta hospitalar?

**Authors:** Wesley Santana Correa-Arruda, Iara dos Anjos Vaez, José Eduardo de Aguilar-Nascimento, Diana Borges Dock-Nascimento

**Affiliations:** 1 Programa de Pós-Graduação em Ciências da Saúde, Universidade Federal de Mato Grosso, Cuiabá, MT, Brasil.

Inicialmente, agradecemos as ponderações realizadas por Rodrigues et al. A ideia central do trabalho foi a de investigar se o jejum noturno, muitas vezes exigido para procedimentos cirúrgicos ou de diagnóstico, impacta na função muscular. Sem dúvida, os achados mostraram que o tempo de jejum noturno, de 8 horas, embora fisiológico, resultou em redução na capacidade funcional determinada pela dinamometria. Isso é agravado pelo fato de o jejum para procedimentos nos hospitais do Brasil, prescrito para 8 horas, na realidade é maior, sendo estimado, em média, em 18 horas, podendo chegar a 24 horas, em alguns casos.^1^Isso é relevante, e os achados mostram que a função muscular pode estar mais prejudicada quando esse jejum prolongado é diurno,^2^diante do paciente acordado e em maior estresse metabólico.^3^Outro ponto importante nos nossos achados é que a força muscular aumentou de acordo com a quantidade do jantar ingerido na noite anterior à aferição da força e após o desjejum e o almoço. Isso mostra a interferência positiva da ingestão de calorias e nutrientes na função muscular. Esses resultados reforçam a necessidade de garantir que o paciente ingira ou receba, via terapia nutricional, toda necessidade nutricional,^4^e que o tempo de jejum para procedimentos, exames ou cirurgias, conforme preconiza o projeto ACERTO,^5^seja reduzido.^6^Assim como o jejum pode resultar em redução da função muscular, levando a piores desfechos clínicos,^7^e a ingestão alimentar melhora essa capacidade muscular, fazem-se necessários, na rotina diária, reduzir o tempo de jejum e garantir maior ingestão de calorias e nutrientes aos pacientes. A inclusão de tabelas e figuras visa apenas dar aos leitores uma visão mais rápida dos achados que, como vocês mesmos registraram, são relevantes. Finalmente, a *odds ratio* também dá uma noção ao leitor da chance de um evento ocorrer, e isso também acrescenta informação − às vezes, maior do que apenas o valor de p. Dentro dessa visão, acreditamos que as conclusões foram coerentes com os objetivos traçados e os resultados obtidos. Esperamos que essas ponderações auxiliem os autores e os leitores a compreenderem melhor o trabalho original publicado pela revista einstein.
